# From Fitting the Average to Fitting the Individual: A Cautionary Tale for Mathematical Modelers

**DOI:** 10.3389/fonc.2022.793908

**Published:** 2022-04-28

**Authors:** Michael C. Luo, Elpiniki Nikolopoulou, Jana L. Gevertz

**Affiliations:** ^1^Department of Mathematics and Statistics, The College of New Jersey, Ewing, NJ, United States; ^2^School of Mathematical and Statistical Sciences, Arizona State University, Tempe, AZ, United States

**Keywords:** cancer, mathematical modeling, personalized therapy, immunotherapy, nonlinear mixed effects modeling

## Abstract

An outstanding challenge in the clinical care of cancer is moving from a one-size-fits-all approach that relies on population-level statistics towards personalized therapeutic design. Mathematical modeling is a powerful tool in treatment personalization, as it allows for the incorporation of patient-specific data so that treatment can be tailor-designed to the individual. Herein, we work with a mathematical model of murine cancer immunotherapy that has been previously-validated against the average of an experimental dataset. We ask the question: what happens if we try to use this same model to perform personalized fits, and therefore make individualized treatment recommendations? Typically, this would be done by choosing a single fitting methodology, and a single cost function, identifying the individualized best-fit parameters, and extrapolating from there to make personalized treatment recommendations. Our analyses show the potentially problematic nature of this approach, as predicted personalized treatment response proved to be sensitive to the fitting methodology utilized. We also demonstrate how a small amount of the right additional experimental measurements could go a long way to improve consistency in personalized fits. Finally, we show how quantifying the robustness of the average response could also help improve confidence in personalized treatment recommendations.

## 1 Introduction

The conventional approach for developing a cancer treatment protocol relies on measuring average efficacy and toxicity from population-level statistics in randomized clinical trials (1–3). However, it is increasingly recognized that heterogeneity, both between patients and within a patient, is a defining feature of cancer (4, 5). This inevitably results in a portion of cancer patients being over-treated and suffering toxicity consequences from the standard-of-care dose, and another portion being under-treated and not benefiting from the expected efficacy of the treatment (6).

For these reasons, in the last decade there has been much interest in moving away from this ‘one-size-fits-all’ approach to cancer treatment and towards personalized therapeutic design (also called predictive or precision medicine) (1, 2, 7). Collecting patient-specific data has the potential to improve treatment response to chemotherapy (6, 8–11), radiotherapy (12–14), and targeted molecular therapy (11, 15–17). However, it has been proposed that personalization may hold the most promise when it comes to immunotherapy (18). Immunotherapy is an umbrella term for methods that increase the potency of the immune response against cancer. Unlike other treatment modalities that directly attack the tumor, immunotherapy depends on the interplay between two complex systems (the tumor and the immune system), and therefore may exhibit more variability across individuals (18).

Mathematical modeling has become a valuable tool for understanding tumor-drug interactions. However, just as clinical care is guided by standardized recommendations, most mathematical models are validated based on population-level statistics from preclinical or clinical studies (19). To truly realize the potential of mathematical models in the clinic, these models must be individually parameterized using measurable, patient-specific data. Only then can modeling be harnessed to answer some of the most pressing questions in precision medicine, including selecting the right drug for the right patient, identifying the optimal drug combination for a patient, and prescribing a treatment schedule that maximizes efficacy while minimizing toxicity.

Efforts to personalize mathematical models have been undertaken to understand glioblastoma treatment response (20, 21), to identify optimal chemotherapeutic and granulocyte colony-stimulating factor combined schedules in metastatic breast cancer (22), to identify optimal maintenance therapy chemotherapeutic dosing for childhood acute lymphoblastic leukemia (9), and to identify optimized doses and dosing schedules of the chemotherapeutic everolimus with the targeted agent sorafenib for solid tumors (23). Interesting work has also been done in the realm of radiotherapy, where individualized head and neck cancer evolution has been modeled through a dynamic carrying capacity informed by patient response to their last radiation dose (24).

Beyond these examples, most model personalization efforts have focused on prostate cancer, as prostate-specific antigen is a clinically measurable marker of prostate cancer burden (25) that can be used in the parameterization of personalized mathematical models. The work of Hirata and colleagues has focused on the personalization of intermittent androgen suppression therapy using retrospective clinical trial data (26, 27). Other interesting work using clinical trial data has been done by Agur and colleagues, focusing on individualizing a prostate cancer vaccine using retrospective phase 2 clinical trial data (25, 28), as well as androgen deprivation therapy using data from an advanced stage prostate cancer registry (29). Especially exciting work on personalizing prostate cancer has been undertaken by Gatenby and colleagues, who used a mathematical model to discover patient-specific adaptive protocols for the administration of the chemotherapeutic agent abiraterone acetate (30). Among the 11 patients in a pilot clinical trial treated with the personalized adaptive therapy, they observed the median time to progression increased to at least 27 months as compared to 16.5 months observed with standard dosing, while also using a cumulative drug amount that was 47% less than the standard dosing (17).

Despite these examples, classically mathematical models are not personalized, but are validated against the average of experimental data. In particular, modelers choose a single fitting methodology, a single cost function to minimize, and find the best-fit parameters to the average of the data. Using the best-fit parameters and the mathematical model, treatment optimization can be performed. Recognizing the limitations of this approach in describing variable treatment response across populations, modelers have begun employing virtual population cohorts (31–33). There is much value in this population-level approach to study variability, but it is not equivalent to looking at individualized treatment response.

In this work, we explore the consequences of performing individualized fits using a minimal mathematical model previously-validated against the average of an experimental dataset. In *Materials and Methods*, we describe the preclinical data collected by Huang et al. (34) on a mouse model of melanoma treated with two forms of immunotherapy, and our previously-developed mathematical model that has been validated against population-level data from this trial (35). Individual mouse volumetric time-course data is fit to our dynamical systems model using two different approaches detailed in *Materials and Methods*: the first fits each mouse independent of the other mice in the population, whereas the second constrains the fits to each mouse using population-level statistics. In *Results*, we demonstrate that the treatment response identified for an individual mouse is *sensitive to the fitting methodology utilized*. We explore the causes of these predictive discrepancies and how robustness of the optimal-for-the-average treatment protocol influences these discrepancies. We conclude with actionable suggestions for how to increase our confidence in mathematical predictions made from personalized fits.

## 2 Materials and Methods

### 2.1 Data Set

The data in this study considers the impact of two immunotherapeutic protocols on a murine model of melanoma (34). The first protocol uses oncolytic viruses (OVs) that are genetically engineered to lyse and kill cancer cells. In (34) the OVs are immuno-enhanced by inserting transgenes that cause the virus to release 4-1BB ligand (4-1BBL) and interleukin (IL)-12, both of which result in the stimulation of the tumor-targeting T cell population (34). The preclinical work of Huang et al. has shown that oncolytic viruses carrying 4-1BBL and IL-12 (which we will call Ad/4-1BBL/IL-12) can cause tumor debulking *via* virus-induced tumor cell lysis, and immune system stimulation from the local release of the immunostimulants (34).

**Figure 1:**
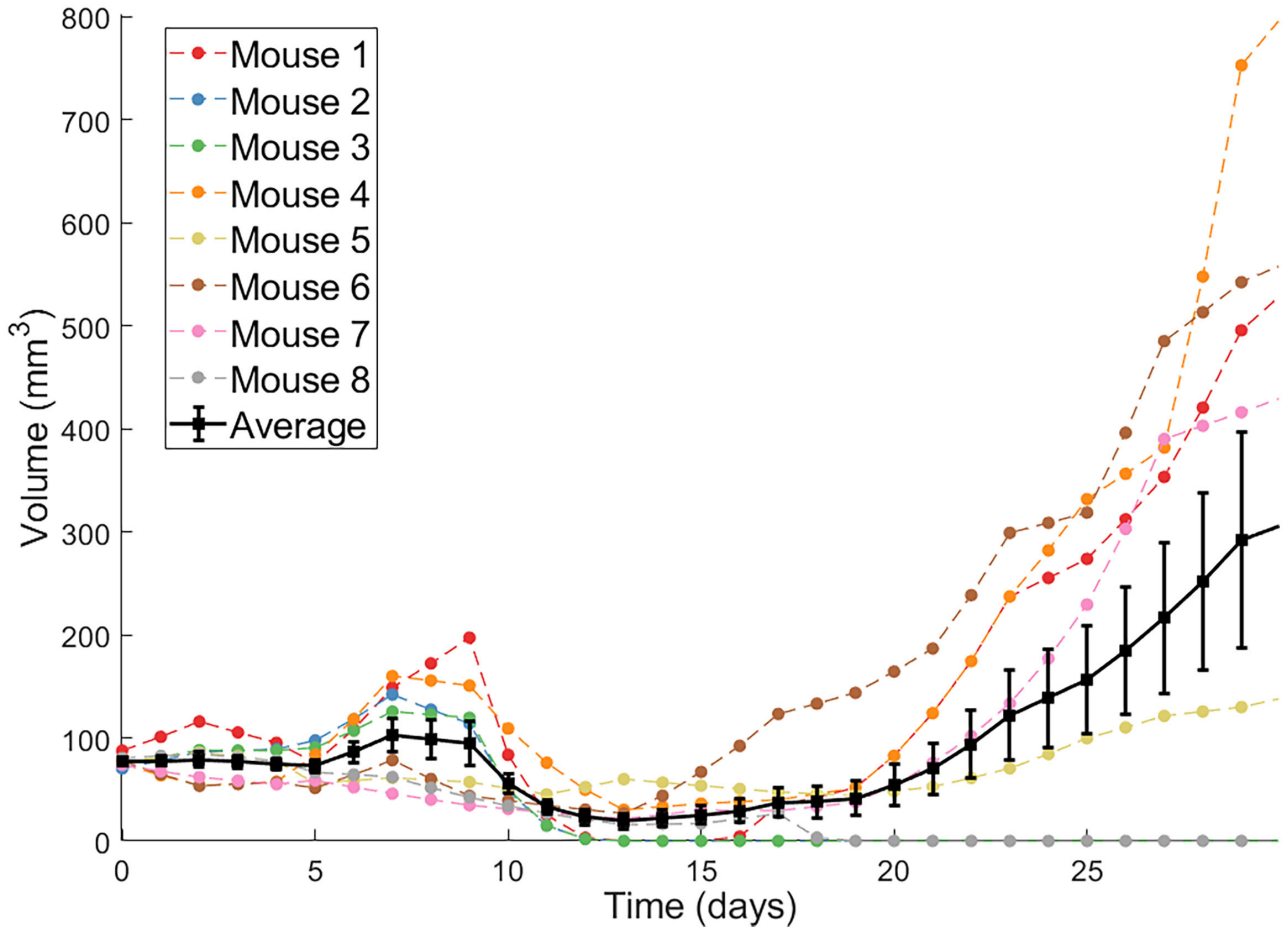
Individual volumetric trajectories are shown for eight mice treated with Ad/4-1BBL/IL-12 (on days 0, 2, 4) + DCs (on days 1, 3, 5). The average, with standard error bars, is also shown in black (34).

The second protocol utilized by Huang et al. are dendritic cell (DC) injections. DCs are antigen-presenting cells that, when exposed to tumor antigens ex vivo and intratumorally injected, can stimulate a strong adaptive immune response against cancer cells (34). Huang et al. showed that combination of Ad/4-1BBL/IL-12 with DC injections results in a stronger antitumor response than either treatment individually (34). Volumetric trajectories of individual mice treated with three doses of Ad/4-1BBL/IL-12 on days 0, 2 and 4, and three doses of DCs on days 1, 3, 5, along with the average trajectory, are shown in **Figure 1**.

### 2.2 Mathematical Model

Our model contains the following five ordinary differential equations:


(1) $$\frac{dU}{dt}=rU-\beta \frac{UV}{N}-\left(\kappa_{0}+c_{kill}I\right)\frac{UT}{N},U\left(0\right)=U_{0},$$

(2) $$\frac{dI}{dt}=\beta \frac{UV}{N}-\delta_{I}I-\left(\kappa_{0}+c_{kill}I\right)\frac{IT}{N},I\left(0\right)=0,$$

(3) $$\frac{dV}{dt}=u_{V}\left(t\right)+a\delta_{I}I-\delta_{V}V,V\left(0\right)=0$$

(4) $$\frac{dT}{dt}=c_{T}I+\chi_{D}D-\delta_{T}T,T\left(0\right)=0,$$

(5) $$\frac{dD}{dt}=u_{D}\left(t\right)-\delta_{D}D,D\left(0\right)=0$$

where *U* is the volume of uninfected tumor cells, *I* is the volume of OV-infected tumor cells, *V* is the volume of free OVs, *T* is the volume of tumor-targeting T cells, *D* is the volume of injected dendritic cells, and *N* is the total volume of cells (tumor cells and T cells) at the tumor site. When all parameters and time-varying terms are positive, this models captures the effects of tumor growth and response to treatment with Ad/4-1BBL/IL-12 and DCs (35). By allowing various parameters and time-varying terms to be identically zero, other treatment protocols tested in Huang et al. (34) can also be described.

This model was built in a hierarchical fashion, details of which have been described extensively elsewhere (32, 35–37). Here, we briefly summarize the full model. Uninfected tumor cells grow exponentially at a rate *r*, and upon being infected by an OV convert to infected cancer cells at a density-dependent rate *βUV/N*. These infected cells get lysed by the virus or other mechanisms at a rate of *δ_I_*, thus acting as a source term for the virus by releasing an average of *α* free virions into the tissue space. Viruses decay at a rate of *δ_v_*. 

The activation/recruitment of tumor-targeting T cells can happen in two ways: 1) stimulation of cytotoxic T cells due to 4-1BBL or IL-12 (modeled through *I*, at a rate of *c_T_*, as infected cells are the ones to release 4-1BBL and IL-12), and 2) production/recruitment due to the externally-primed dendritic cells at a rate of χ*_D_*. These tumor-targeting T cells indiscriminately kill uninfected and infected tumor cells, with the rate of killing that depends on IL-12 and 4-1BBL production (again, modeled through *I* in the term (*κ*_0_ + *c_κill_I*)), and they can also experience natural death at a rate of *δ_T_*. The time-dependent terms, *u_v_* (*t*) and *u_D_* (*t*), represent the source of the drug and are determined by the delivery and dosing schedule of interest.

### 2.3 Fitting Methodologies

For both fitting methodologies, the full set of model parameters {*r*,*β*,*α*,*δ_v_*,*ĸ*_0_,*δ_T_*,χ*_D_*,*δ_I_*,*c_ĸill_*,*c_T_*,*δ_D_*,*U*_0_}, which includes the initial uninfected tumor volume, is fit to each individual mouse.

#### 2.3.1 Independently Fitting Individuals

Our first attempt at individualized fitting is to find the parameter set that minimizes the square of the *ℓ*^2^-norm between the model and the individual mouse data:


(6) $$\zeta =\sum_{t=0}^{n}{\left(V_{model}\left(t\right)-V_{data}\left(t\right)\right)}^{2}$$

where *V_model_* (*t*) = *U*(*t*) + *I*(*t*) is the volumetric output predicted by our model in eqns. (1)-(5), *V_data_* (*t*) represents the volumetric data for an individual mouse, and *n* is the last time point at which the volume is measured in the experiments.

To independently fit an individual mouse, 12-dimensional space is first quasi-randomly sampled (with each point sampled in the range [0,1]) using high-dimensional Sobol’ Low Discrepancy Sequences (LDS). LDS are designed to give rise to quasi-random numbers that sample points in space as uniformly as possible, while also (typically) having faster convergence rates than standard Monte Carlo sampling methods (38). Each randomly sampled point is then scaled to be in a biologically plausible range for the corresponding parameter value. For those parameters that were previously-fit to the average of the experimental data (*r*,*β*,χ*_D_*,*c_T_*,*c_ĸill_*), the range was set using the lower and upper-bound of the 95%-credible interval for the parameter, as determined in (35). For parameters not fit to the average in prior work, the minimum and maximum values were set to 50% and 200% of the value the parameter was fixed to for the average, respectively. See **Supplementary Table 1** for details.

After the best-fit parameter set has been selected among the 10^6^ randomly sampled sets chosen by LDS, the optimal is refined using simulated annealing (39). Having observed that the landscape of the objective function near the optimal parameter set does not contain local minima, we randomly perturb the LDS-chosen parameter set, and accept any realistic parameter changes that decrease the value of the objective function - making the method equivalent to gradient descent. We consider a parameter set realistic at this stage if all parameter values are non-negative. This random perturbation process is repeated until no significant change in ζ can be achieved, which we defined as the relative change in *ζ* for the last five accepted parameter sets being less than 10^–5^. We call this final parameter set the optimal parameter set. More details can be found in **Supplementary Algorithm 1**.

It is important to note that, by approaching fitting in this way, the parameters for Mouse *i* depend only the volumetric data for Mouse *i*; that is, the volumetric data for the other mice are not accounted for.

#### 2.3.2 Fitting Individuals with Population-Level Constraints

Nonlinear mixed effects (NLME) models incorporate fixed and random effects to generate models to analyze data that are non-independent, multilevel/hierarchical, longitudinal, or correlated (40). Fixed effects refer to parameters that can generalize across an entire population. Random effects refer to parameters that differ between individuals that are randomly sampled from a population. To employ NLME for our mathematical model, for each mouse *i* we define the structural model *T*(*t_ij_*,ψ*_i_*)=*U*(*t_j_*)+*I*(*t_j_*). We assume that each parameter ψ*_i,k_* in the parameter set ψ*_i_* is lognormally distributed with mean ${\bar{\psi }}_{i,k}$ and standard deviation *ω_i,k_*:


(7) $$log\left(\psi_{i,k}\right)∼𝒩\left(log\left({\bar{\psi }}_{i,k},\omega_{i,k}^{2}\right)\right)$$

We also assume that the error is a scalar value proportional to our structural model. Our resultant mixed effects model is:


(8) $$y_{ij}=T\left(t_{i,j},\psi_{i}\right)+bT\left(t_{i,j},\psi_{i}\right)\varepsilon_{i,j},i=1,\ldots ,M,j=0,\ldots ,n_{i}-1,$$

where *y_ij_* is the predicted tumor volume at each day *j* for each individual *i* (that is, at time *t_ij_*), *M* = 8 is the number of mice, *n_i_* = 31 is the number of observations per mouse, and *ε_ij_* is the random noise term, which we assume to follow a standard normal distribution.

Typically, NLME models attempt to maximize the likelihood of the parameter set given the available data. There does not exist a general closed-form solution to this maximization problem (41), so numerical optimization is often needed to find a maximum likelihood estimate. In this work, we employ Monolix (42), which uses a Markov Chain Monte Carlo method to find values of the model parameters that optimize the likelihood function. To implement NLME in Monolix, we first processed and arranged our experimental data consisting of tumor volume and dosing schedule in a Monolix-specified spreadsheet. The data is then censored to avoid overfitting very small tumor volumes, as detailed in (43). To understand why this overfitting occurs in uncensored data, consider the scenario where the model predicts a volume 10^–4^ mm^3^ at a time point whereas the experimental measurement is 0 mm^3^. The parameter set corresponding to this prediction is assigned a lower likelihood, despite the fact that 10^–4^ is a perfectly reasonable model prediction of an experimental measurement of 0. To avoid penalizing insignificant prediction errors at very small tumor volumes, the data has been censored so that when the negative log likelihood is computed, instead of calculating the likelihood the model gives exactly the value of 0, it computes the likelihood the model predicts a value between 0 and 1. While this censoring was necessary to prevent NLME from over-fitting data points of volume zero at the expense of the fits to the nonzero data points, such censoring was not required for the independent fitting approach, as there we are just minimizing the sum of the square error. That is, in the independent fitting approach, when the model predicts a very small volume and the experimental measurement is 0, the contribution to the sum of the square error is negligible and thus censoring is not needed.

In order to solve this NLME model in Monolix, initial guesses are needed for the mean and standard deviation of our lognormally-distributed parameters. Based on previous fits to the average of the data in (37), we used the following set of initial guesses for the mean of each parameter:


$$\left[r,\beta ,\alpha ,\delta_{v},\kappa_{0},\delta_{T},\chi_{D},\delta_{I,}c_{kill},c_{T},\delta_{D},U_{0}\right]=[0.32,1,3,2.3,2,0.35,5.5,1,0.51,1.2,0.35,55.6],$$

and after numerical exploration, we ended up choosing the initial standard deviations as:


$$\left[\omega_{r},\omega_{\beta },\omega_{\alpha },\omega_{\delta_{V}},\omega_{\kappa_{0}},\omega_{\delta_{T}},\omega_{\chi_{D}},\omega_{\delta_{I}},\omega_{C_{kill}},\omega_{C_{T}},\omega_{\delta_{D}},\omega_{\kappa_{0}}\right]=\left[0.25,0.5,1,0.1,1,0.1,0.25,0.1,0.5,0.5,0.1,5\right]$$

### 2.4 Practical Identifiability *via* the Profile Likelihood Method

It is well-established that estimating a unique parameter set for a mathematical model can be challenging due to the limited availability of often noisy experimental data (44). A non-identifiable model is one in which multiple parameter sets give “good” fits to the experimental data. Here, we will study the practical identifiability of our system in eqns. (1) - (5) using the profile likelihood approach (45, 46).

A single parameter is profiled by fixing it across a range of values, and subsequently fitting all other model parameters to the data (44). To execute the profile likelihood method, let *p* be the vector that contains all parameters of the model, *θ* be one parameter of interest contained in the vector *p*. The profile likelihood *PL* for the parameter *θ* is defined in (47) as:


(9) $$PL\left(\theta \right)=\underset{p\in \{p|p_{k}=\theta \}}{\min}\left({\sum_{t=0}^{n}\left(\frac{V_{data}\left(t\right)-V_{model}\left(t;p\right)}{\sigma \left(t\right)}\right)}^{2}\right)$$

where *V_model_* (*t*;*p*) = *U*(*t*) + *I*(*t*) is the volumetric output predicted by our model for parameter set *p*, and ${\bar{V}}_{data}$ represents the average volume across all mice at that time point with corresponding standard deviation *σ*(*t*). For normally distributed observational noise this corresponds to the maximum likelihood estimate of *θ*:


(10) $$PL(\theta )=\underset{p\in \{p|p_{k}=\theta \}}{\min}\left(-2LL(p;V_{data}(0),\text{...},V_{data}(n))\right)$$

where *LL*(*p*;*V_data_* (0)*,...,V_data_* (*n*)) is the log of the likelihood function. The likelihood function represents the likeliness of the measured data *V_data_* given a model with parameters *p* (48). This likelihood is higher for a parameter set that is more likely given the available data, and it is smaller for parameter sets that are less likely given the data. The profile likelihood curve for any parameter of interest *θ* is found using the following process:

1. Determine a range for the parameter values of *θ*.
2. Fix *θ* = θ^*^ at a value in the range.
3. Find the value of the non-fixed parameters that minimize the objective function in eqn. (9). The quasi-random Monte Carlo method with gradient descent was used for the fitting, as detailed previously.
4. Evaluate the objective function at those optimum values for the fixed value of θ^*^.
5. Repeat the process described in steps 2-4 for a discrete set of values in the range of the parameter *θ*. This yields the profile likelihood function for the parameter *θ*.

This process results in a population-level (not individual) profile likelihood curve for each parameter. Once *PL*(*θ*) is determined, the confidence interval for *θ* at a level of significance *α* can be computed using:


(11) $$PL\left(\theta \right)-2LL\left(p_{k}^{*}\right)\leq \Delta_{\alpha }$$

where ∆*_α_* denotes the *α* quantile of the *χ*^2^ distribution with *df* degrees of freedom (which represents the number of fit model parameters when calculating *PL*(*θ*)) (44). We use *α* = 0.95 for a 95% confidence interval. The intersection points between the threshold $2LL\left(p_{k}^{*}\right)+\Delta_{\alpha }$ and *PL*(*θ*) result in the bounds of the confidence interval. A parameter is said to be practically identifiable if the shape of the profile likelihood plot is close to quadratic on a finite confidence interval (49). Otherwise, a parameter is said to be practically unidentifiable.

## 3 Results

### 3.1 Personalized Fits

**Figure 2:**
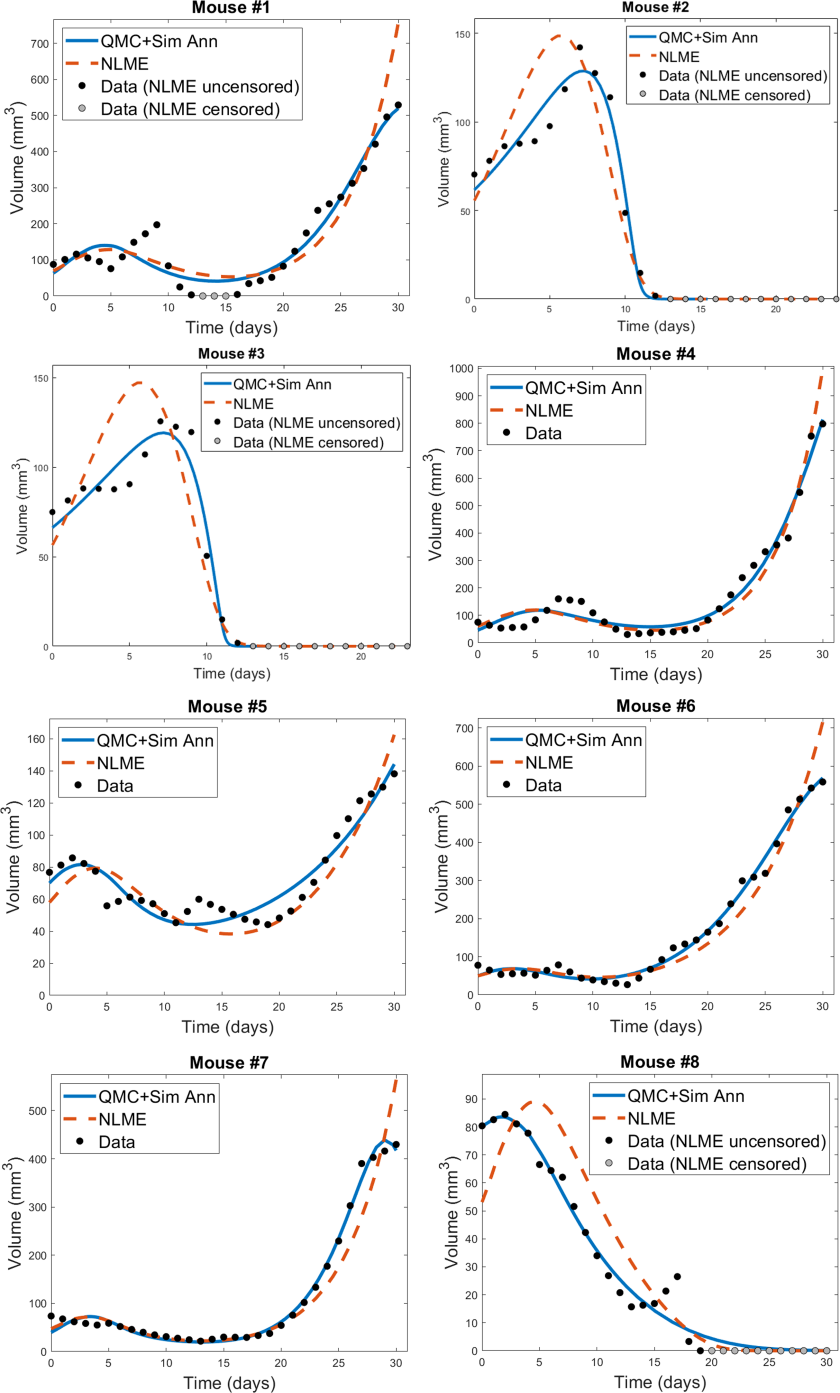
Best-fit for each mouse treated with Ad/41BBL/IL-12 and DCs in the order VDVDVD at a dose of 2.5 × 10^9^ OVs and 10^6^ DCs (34). The QMC fits (in which each mouse is treated independently of the others) are shown in blue, and the NLME fits are shown in red. The experimental data (black if uncensored for NLME fitting, grey if censored) is also provided on each plot.

The individual mouse data in response to treatment with Ad/4-1BBL/IL-12 + DCs (34) is fit using the two methodologies discussed previously: 1) quasi-Monte-Carlo method with gradient descent in which each mouse is fit independently (which we will call the “QMC” method for short), and 2) nonlinear mixed effects modeling in which population-level statistics constrain individual fits. In **Figure 2**, we can see the best-fit for each mouse using the two fitting approaches.

We do observe some shortcomings in the fits, particularly at earlier time points. These are most-pronounced in Mouse 1 and 4, where the model cannot capture the early-time decrease in tumor volume. This highlights that a model validated against the average of a dataset may not be fully sufficient at describing individual trajectories. That said, we overall find that the model is able to capture the trends in the volumetric data despite the heterogeneity across individual mice. While a more detailed model could potentially pick up some early-time trends our model did not capture, this would come at the expense of introducing more (likely non-identifiable) parameters.

For each mouse, the QMC algorithm results in a fit that more accurately captures the dynamics in the experimental data. The differences between the two fitting methodologies explain why this is occurring. NLME assumes each parameter is sampled from a lognormal distribution whose mean and variance are determined by the full population of mice. The estimated lognormal distributions for each model parameter are shown in **Figure S1**. On the other hand, the QMC algorithm fits each mouse independently, and despite the initial bounds set on the parameters when sampling parameter space, gradient descent relaxes these constraints and the end result is that non-negativity is the only constraint imposed. This allows the QMC algorithm to explore a larger region of parameter space, resulting in better fits. The potential downside, as we will show, is that the QMC algorithm can select parameter values that deviate more significantly from the average value. This variability may represent the true variability across individual mice, or may be a consequence of doing independent fits.

**Figure 3:**
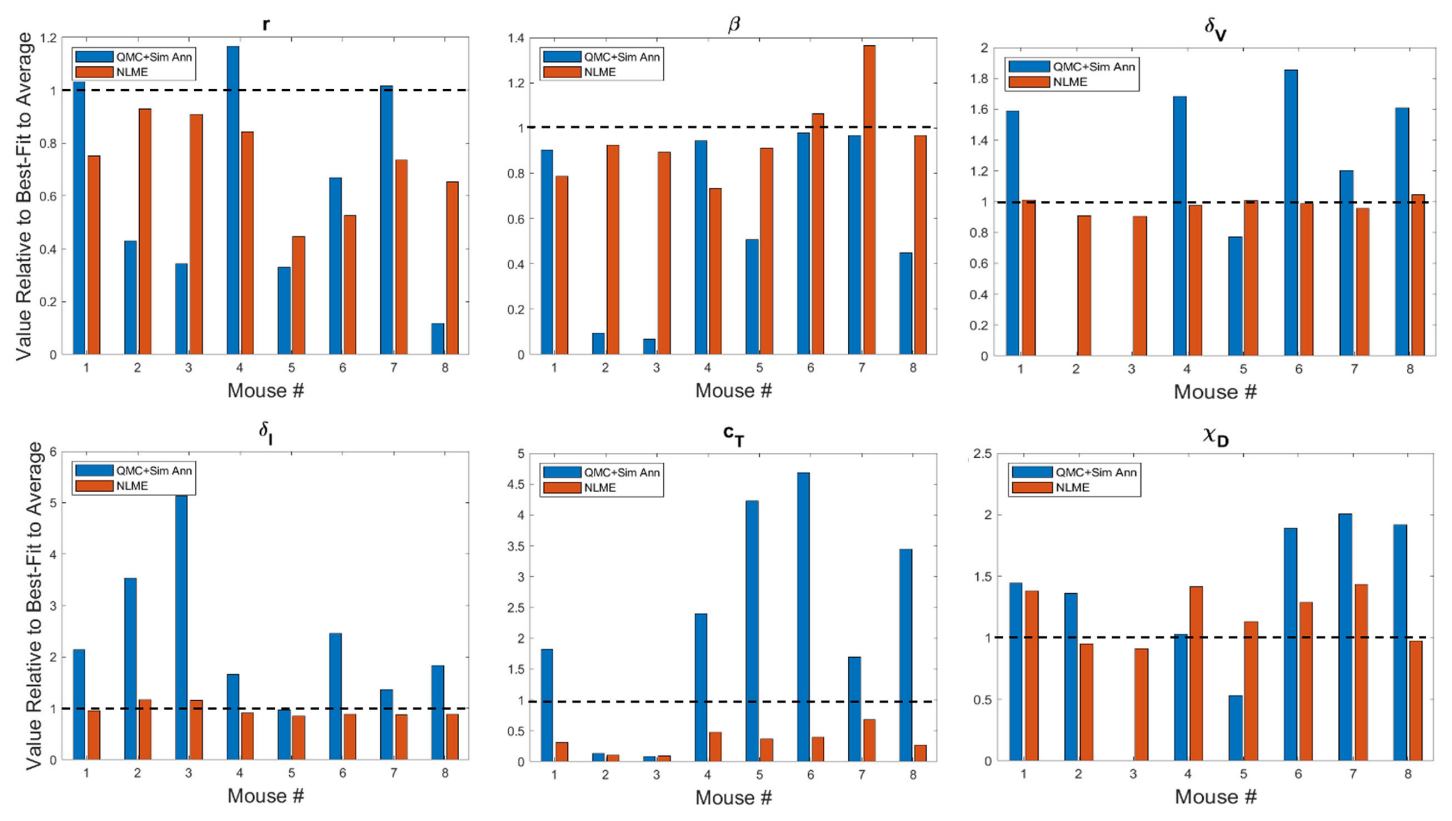
Best-fit values of tumor growth rate parameter *r*, virus infectivity parameter *β*, viral decay rate *δ_v_*, infected cell lysis rate *δ_I_*, T cell stimulation term by immunostimulants *c_T_*, and T cell stimulation term by DCs *χ_D_*. The best-fit values are shown for each mouse and are presented relative to the best-fit value of the parameter in the average mouse (35). Therefore, a value of 1 (shown in the dashed black line) means the parameter value is equal to that in the average mouse, less than 1 is a smaller value, and greater than 1 is a larger value. Values for other model parameters are shown in **Figure S2**.

In **Figure 3** and **Figure S2** we show the best-fit parameter value for each mouse and fitting methodology relative to the best-fit parameter value for the average mouse. For example, the best-fit value of the tumor growth rate *r* to the average of the control data has been shown to be *r* = 0.3198 (35). Since Mouse 1 has a relative value of 1.0916 when fitting is done using QMC, the value of *r* predicted for that Mouse is 9.16% larger than the value for the average mouse, meaning QMC predicts *r* = 0.3491 for Mouse 1. On the other hand, the relative value is 0.7512 when fitting is done using NLME, meaning the predicted value is *r* = 0.2402, which is 24.88% less than the value for the average mouse.

A study of the values a parameter can take on across methodologies reveals that while most values are of the same order of magnitude, differences can exist across methodologies. As expected due to the constraining lognormal distribution, NLME-associated parameters exhibit smaller variations from the best-fit parameter for the average mouse than QMC-associated parameters. Generally speaking, the variation seen could be explained by a heterogeneous response to the treatment protocol across mice. For instance, a very small value of *χ_D_* in Mouse 3 indicates the DCs are not successfully stimulating the production of tumor-targeting T cells. As another example, a very small value of *κ*_0_ in Mouse 2 indicates that in the absence of immunostimulation, the T cells are unable to target and destroy cancer cells. There is one scenario that emerges in both Mouse 2 and 3, however, that cannot be explained by a heterogeneous treatment response. In particular, the QMC approach predicts that these mice have *δ_v_* = 0, indicative that the virus will not decay over the 30-day experimental time period. As this scenario is highly unlikely, we also refit these mice using the QMC approach, assuming a (somewhat arbitrary) lower bound on the viral decay rate of *δ_v_* = 0.46 day^–1^, which assumes the decay rate can never be smaller than a quarter of the average value of *δ_v_* = 2.3 day^–1^ (37). Refitting both mice with the QMC algorithm and this additional constraint resulted in the best-fit value of *δ_v_* being this strict lower bound. All treatment predictions presented in this manuscript were identical whether Mouse 2 or 3 was analyzed using the parameter set with *δ_v_* = 0 or the parameter set where *δ_v_* was a quarter the maximum value.

Looking across methodologies, parameter disparities are the most pronounced in *c_T_*, the rate of cytotoxic T cell stimulation from 4-1BBL and IL-12. The QMC-predicted parameters cover a much larger range of values relative to the average mouse. According to the QMC fits, *c_T_* can range anywhere from 92.15% below the value in the average mouse to 4.69 times higher than the value in the average mouse. Compare this to the NLME-predicted values of *c_T_*, which can range from 90.29% below the value in the average mouse to 31.87% below the value for the average mouse. What is clear from looking at the best-fit parameter values across methodologies is that it is not differences in a single or small set of parameter values that explain the difference in fits. The nonlinearities in the model simply do not allow the effects of one parameter to be easily teased out from the effects of the other parameters.

### 3.2 Personalized Treatment Response at Experimental Dose

**Figure 4:**
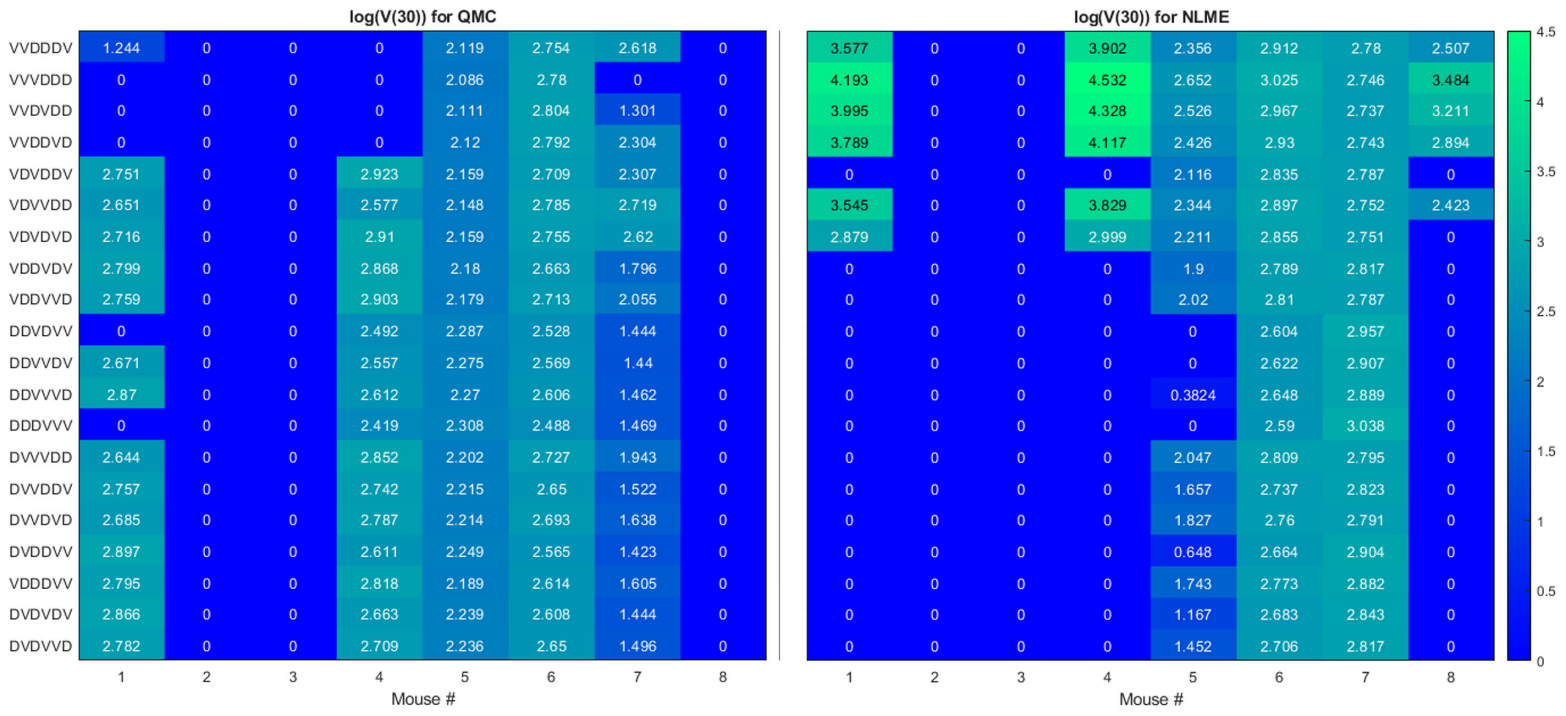
Heatmaps showing the log of the tumor volume measured at 30 days, at the OV and DC dose used in (34). If log(*V* (30))≤1, its value is shown as 0 on the heatmap. Left shows predictions when parameters are fit using QMC, and right shows NLME predictions.

Here we seek to determine if the two sets of best-fit parameters for a single individual yield similar personalized predictions about tumor response to a range of treatment protocols. The treatment protocols we consider are modeled after the experimental work in (34). Each day consists of only a single treatment, which can be either an injection of Ad/4-1BBL/IL-12 at 2.5 × 10^9^ viruses per dose, or a dose of 10^6^ DCs. Treatment will be given for six consecutive days, with three days of treatment being Ad/4-1BBL/IL-12, and three days being DCs. If only one dose can be given per day, there are exactly 20 treatment protocols to consider. The 20 protocols are shown on the vertical axis in **Figure 4**, where *V* represents a dose of Ad/4-1BBL/IL-12, and *D* represents a dose of dendritic cells.

To quantify predicted tumor response, we will simulate mouse dynamics using the determined best-fit parameters for each of the 20 6-day protocols. Unless otherwise stated, we will use the predicted tumor volume after 30 days, *V(30*), to quantify treatment response. For each fitting methodology, mouse, and protocol we display the log (*V*(30)) in a heatmap (as in **Figure 4**). For all *V(30*)≤1 mm^3^, we display the logarithm as 0, as showing negative values would hinder cross-methodology comparison and overemphasize insignificant differences in treatment response. We consider any tumor with *V*(30)<1 mm^3^ to be effectively treated by the associated protocol. Any nonzero values correspond to the value of log(*V*(30)) when *V*(30)>1 mm^3^, and we assume these tumors have not been successfully treated. The resulting heatmap at the experimental dose of 2.5 × 10^9^ viruses per dose, and 10^6^ DCs per dose is shown in **Figure 4**.

Ideally, we would find that treatment response to a protocol for a given mouse is independent of the fitting methodology utilized, at least in the binary sense of treatment success or failure. However, that does not generally appear to be the case for our data, model and fitting methodologies, as we elaborate on here.

**Figure 5:**
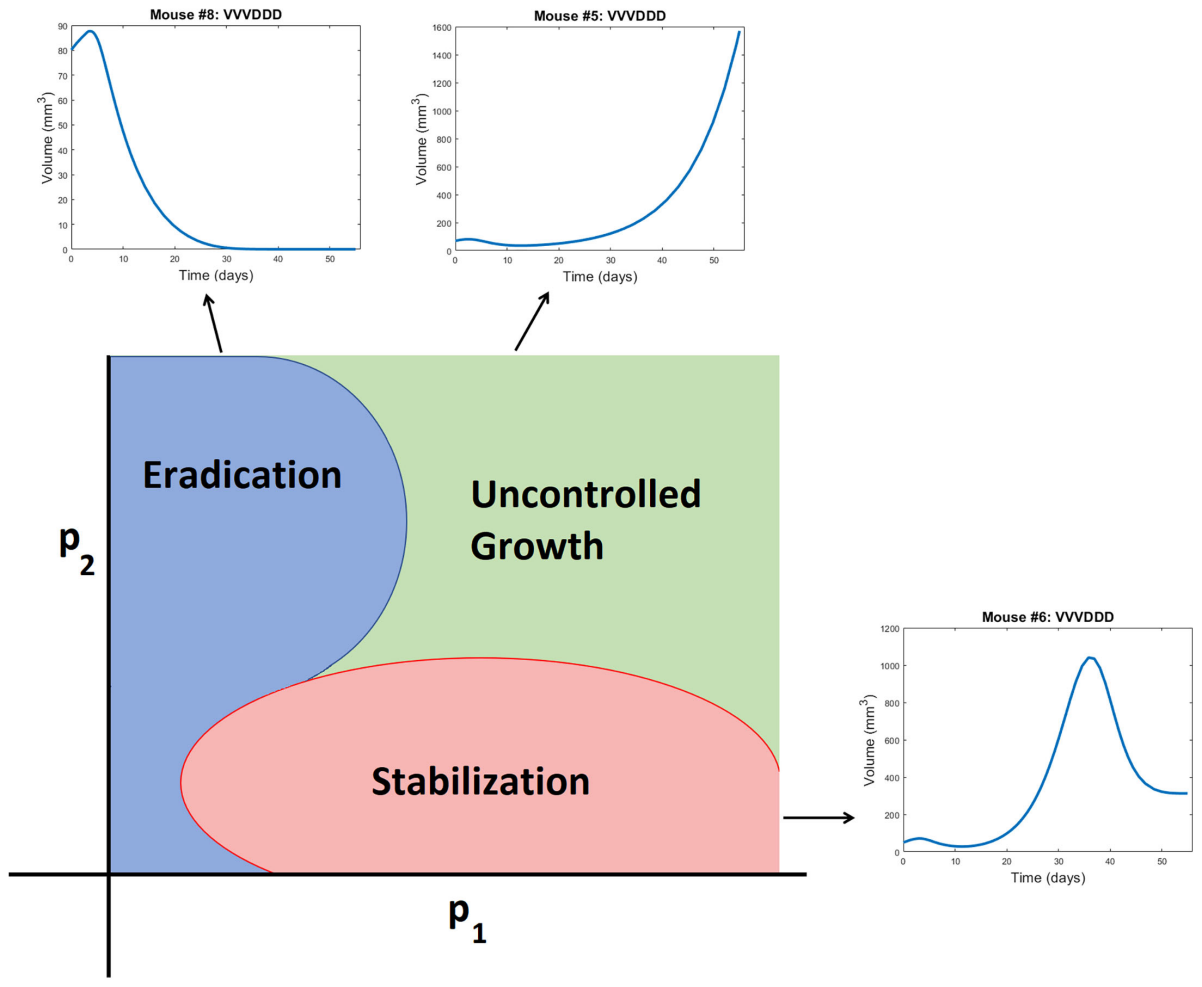
Schematic representation of a bifurcation diagram in two-dimensional parameter space. For certain nonlinear combinations of parameters, a treatment can successfully eradicate a tumor (as occurs for Mouse 8 treated with VVVDDD according to NLME parameters), result in tumor stabilization (as occurs for Mouse 6 treated with VVVDDD according to NLME parameters), or can fail to control the tumor (as occurs for Mouse 5 treated with VVVDDD according to NLME parameters). Note the bifurcation diagram is dependent on both the dose of drug being given, and the ordering of those drugs.

- **Cumulative statistics on consistencies across methodologies.** As shown in **Figure 4**, the two fitting methodologies give the same qualitative predictions for 73.75% (118/160) of the treatment protocols. Of the 118 agreements, 57 consistently predict treatment success whereas 61 consistently predict treatment failure. It is of note that these numbers only change slightly if we use *V(80*) as our measurement for determining treatment success or failure (81.875% agreement with 78/131 consistently predicting eradication and 53/131 consistently predicting failure - see **Figure S3**). Mouse 2, 3 and 6 have perfect agreement across fitting methodologies, and Mouse 7 has 95% agreement across methodologies. For these mice, treatment response is generally not dependent on dosing order. For instance, Mouse 2 and 3 are successfully treated by all twenty protocols considered, whereas Mouse 6 cannot be successfully treated by any protocol. In fact, *V(30*) for Mouse 6 is highly conserved across dosing order, suggesting that the ordering itself is having minimal impact on treatment response. While performing a bifurcation analysis in 11D parameter space is not feasible, what is clear is that for the mice with significant agreement across methodologies, the best-fit parameters must be sufficiently far from the bifurcation surface, as shown in the schematic diagram in **Figure 5**. As a result, predicted treatment response is not sensitive to changes in the parameter values that result from using a different fitting methodology. While not equivalent, they also do not appear to be sensitive to dosing order.
- **Cumulative statistics on inconsistencies across methodologies.** The two fitting methodologies give different qualitative predictions for 26.25% (42/160) of the treatment protocols (see **Figure 4**). Mouse 1 and 4 are largely responsible for these predictive discrepancies, with Mouse 1 having inconsistent predictions for 75% of protocols, and Mouse 4 having inconsistent predictions for 90% of protocols. Note that each methodology must agree for the protocol VDVDVD, as this was the experimental protocol that was used for parameter fitting. So, 95% is the maximum disagreement rate we can see across methodologies for a given mouse. We observe that the QMC-associated parameter set is much more likely to predict treatment failure for these mice, whereas the NLME parameter set is more likely to predict treatment success. Contrary to the mice for which there is significant cross-methodology agreement, we see a high dependency of treatment response to dosing order for Mouse 1 and 4. From the perspective of the high dimensional bifurcation diagram, these parameters must fall sufficiently close to the bifurcation surface so that parametric changes that result from using different fitting methodologies can lead to wildly different predictions about treatment response (see schematic in **Figure 5**). In turn, this appears to make these mice significantly more sensitive to dosing order.

Though the results in this paper are presented for one best-fit parameter set per methodology, we have also explored how parametric uncertainty influences treatment predictions. In particular, for the QMC fitting method, for each mouse we identified suboptimal parameter sets by performing Sobol sampling in a 10% range about the optimal parameter set. Any sampled parameter set that gives a goodness-of-fit within 10% of the optimal is considered a suboptimal parameter set (see **Figure S4**). For all such suboptimal parameter sets, treatment response to the 20 protocols was determined. This allows us to study if binary treatment response is insensitive to the precise best-fit parameters used. In **Figure S5** we show the probability a treatment is effective for each mouse across all suboptimal parameter sets. Overwhelmingly, treatment response predicted for an individual mouse and protocol shows excellent agreement across suboptimal parameter sets. Besides treatment response to the protocols VDVVDD and VDVDVD for Mouse 7, predicted treatment response across suboptimal parameter sets agrees over a minimum of 95% of the suboptimal parameter sets. This is seen in **Figure S6** by the probabilities of an effective treatment being either >0.95 or <0.05. As small parametric perturbations that result in “good” fits to the data do not significantly influence predicted treatment response, we conclude it is reasonable to compare the prediction across methodologies using only the best-fit parameters.

### 3.3 Exploring Predictive Discrepancies Between Fitting Methodologies

The predictive discrepancies across fitting methodologies begs the question of whether the parameters we are fitting are actually practically identifiable given the available experimental data. To explore this question, we generated profile likelihood curves for fitting the *average* tumor growth data, following the methodology detailed in Section 2. As a first step, we fixed the parameters whose values we could reasonably approximate from experimental data: *δ_I_* = 1, *α* = 3000, *δ_v_* = 2.3, *ĸ*_0_ = 2, *δ_T_* = 0.35, and *δ_D_* = 0.35 (37). This means we are using *df* = 5 in the calculation of the threshold, as the generation of each profile likelihood curve requires fitting four model parameters plus the initial condition *U(0*).

**Figure 6:**
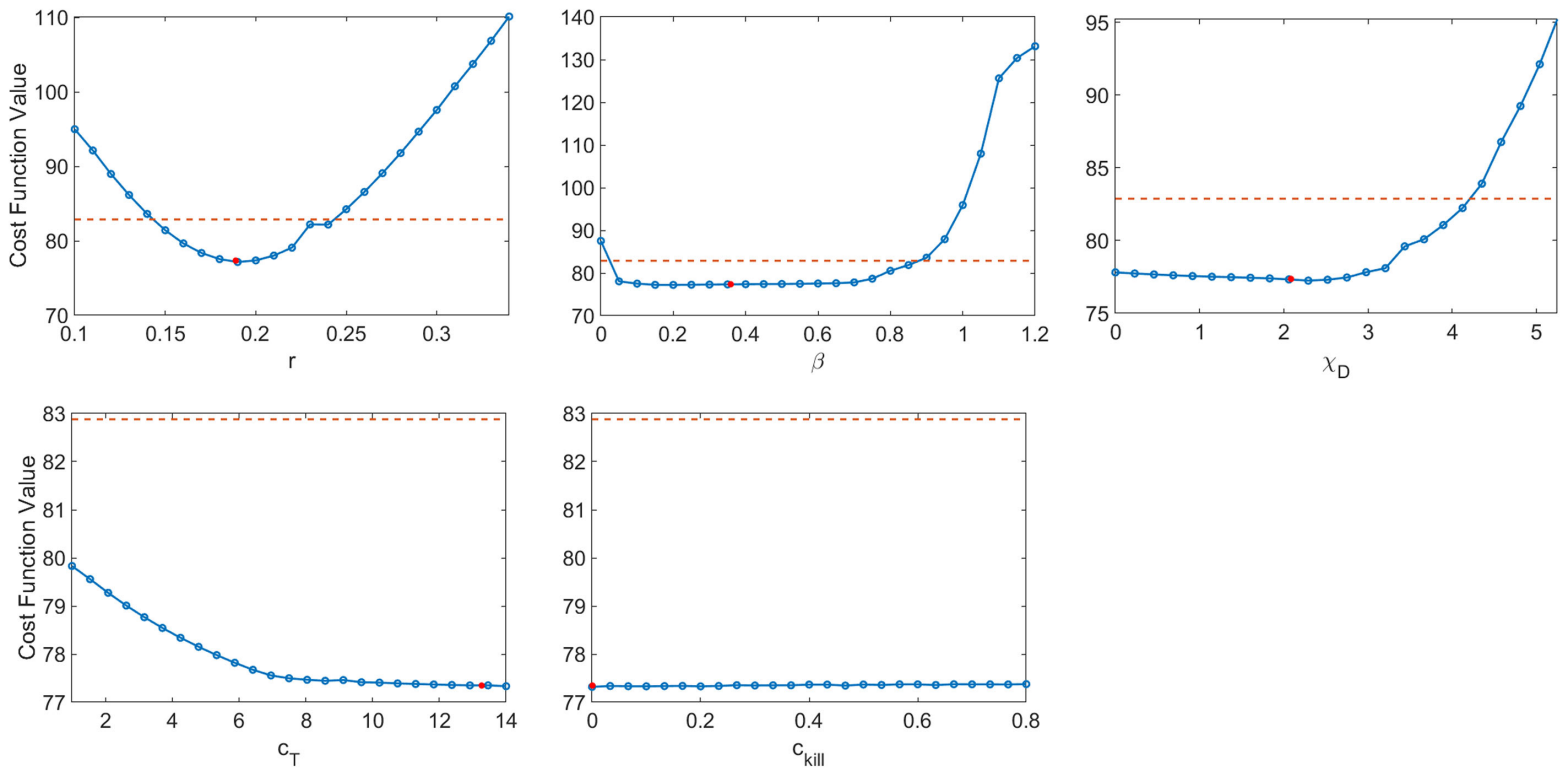
Profile likelihood curves. Top row: tumor growth rate *r*, infectivity rate *β*, T cell activation rate by DCs *χ_D_*. Bottom row: T cell stimulation rate by immunostimulants *c_T_*, and rate at which immunostimulants enhance cytotoxicity of T cells *c_ĸill_*. The threshold (red dashed line) is calculated using *df* = 5 and a 95% confidence interval.

The resulting profile likelihood curves in **Figure 6** show that, even under the assumption that six of the eleven non-initial condition parameters are known, several of the fit model parameters lack practical identifiability. The tumor growth rate *r* and the infectivity parameter *β* are both practically identifiable, ignoring slight numerical noise. The T cell activation parameters *χ_D_* and *c_T_* lack practical identifiability as they have profiles with a shallow and one-sided minimum (44). The profile for *c_kill_* demonstrates that the model can equally well-describe the data over a large range of values for this enhanced cytotoxicity parameter. The flat likelihood profile is indicative of (local) structural unidentifiability, which also results in the parameter being practically unidentifiable (44). It is worth noting that the original work fitting to the average mouse was done in a *hierarchical* fashion (35, 37), and this circumvented the identifiability issues that emerge when doing simultaneous parameter fitting.

As we are unable to exploit the benefits of hierarchical fitting when performing personalized fits, this lack of practical identifiability poses significant issues for treatment personalization. We have already seen the consequences of this when we observed that despite both giving good fits to the data, QMC and NLME make consistent qualitative predictions in only 73.75% of the treatment protocols tested across all individuals. While the lack of practical identifiability helps explain why this can happen, it does not explain the mechanisms that drive predictive differences. To this end, we will now focus on the simulated dynamics of Mouse 4 in more detail, as this was the mouse with the most predictive discrepancies across methodologies.

**Figure 7:**
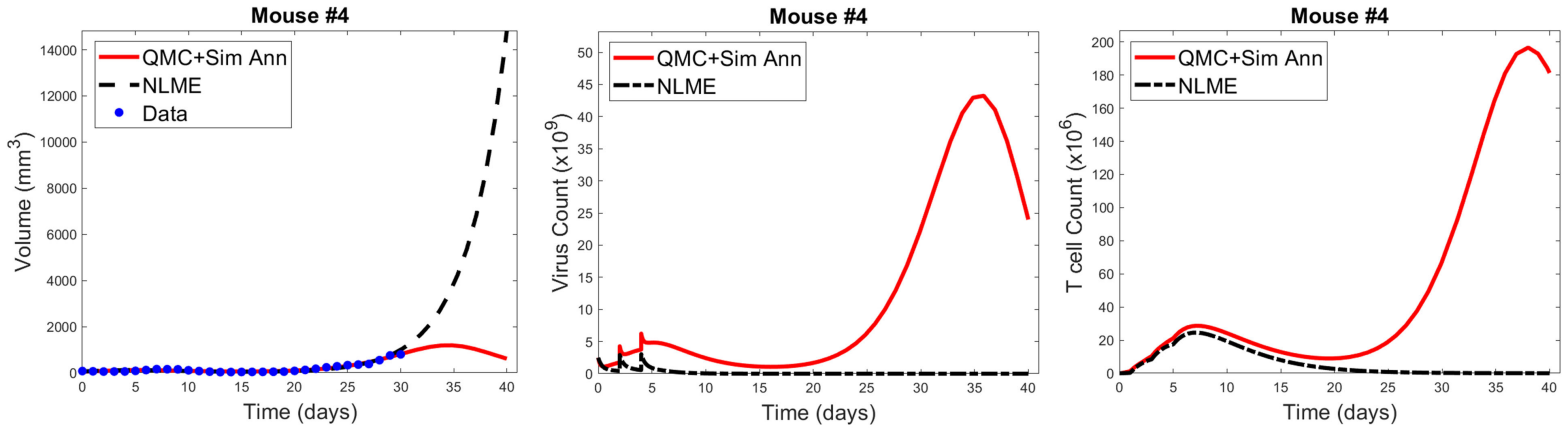
Left: QMC and NLME-associated fits to Mouse 4 treated with VDVDVD, with model predictions extended 10 days beyond the data-collection window. Center and right: Predicted virus and T cell counts associated with each fitting methodology, respectively.

As shown in **Figure 7**, when we simulate the model ten days beyond the data-collection window, we see that the QMC and NLME parameters fall on different sides of the bifurcation surface. In particular, in the QMC-associated simulation, at around 34 days the tumor exhibits a local maximum in volume and continues to shrink from there (**Figure 7**, left). This is in comparison to the NLME-associated simulation, where the tumor grows exponentially beyond the data-collection window. To uncover the biological mechanism driving these extreme differences, we look at the “hidden” variables in our model - that is, variables for which we have no experimental data. As shown in **Figure 7**, despite the similar fits to the volumetric data, the two parameters sets predict drastically different dynamics for the OVs and T cells. For the NLME-associated parameters, the virus and T cell population die out, eventually resulting in unbounded tumor growth. On the other hand, the virus and T cell population remain endemic throughout the simulation when using the QMC-associated parameters, driving the tumor population towards extinction.

It is common knowledge that more data improves parameter identifiability. Not all data is created equal, however. We could get a lot more time-course data on total tumor volume over the 30-day window, but that would not necessarily improve parameter identifiability. Instead, we have identified that the addition of a single data point, for the right variable, at the right time, could go a long way in reconciling predictive discrepancies across fitting methodologies. To make this concrete, suppose we had data that, for Mouse 4, no tumor-targeting T cells are detected at 30 days. If we introduced a modified cost function that weighed both the contribution of the tumor volume and this T cell measurement, the parameter set identified by QMC would no longer be optimal, as it predicts a T cell burden on the order of 10^8^ (100×10^6^). The optimal parameter set should be one for which *T*→0, and once this occurs, there is no mechanism to control the tumor in the long-term. As a result, the tumor must regrow, just as predicted for the NLME-associated parameters. While this thought experiment does not suggest all practical identifiability issues would be reconciled by having this one data point, it does indicate why the predictive discrepancies we see for Mouse 4 (and also Mouse 1) would be at least partially resolved by the addition of a single data point on tumor-targeting T cell volume. This highlights that although one must be quite cautious in using mathematical models to make personalized predictions, models can help us determine precisely what additional data is needed so that we can have more trust in our mathematical predictions.

### 3.4 Personalized Treatment Response to the Optimal for Average Protocol

Ideally, when an optimal prediction is made for the average of a population, that optimal treatment protocol would also well-control the tumors of individual patients in the population. However, it is well known and supported by our earlier work with virtual populations that this is not necessarily the case. In (32) we showed that the experimental dose being considered herein is *fragile or non-robust*. We define a dosing regime as robust if virtual populations that deviate somewhat from the average population have the same qualitative response to the optimal-for-the-average protocol. Otherwise, a protocol is called fragile. The ability to classify fragility/robustness relies on the generation of a virtual population cohort that mimics a broad spectrum of individuals with different disease dynamics (31–33). By determining treatment response for each individual in the virtual cohort, we arrive at a statistic describing the likelihood the considered treatment is effective across heterogeneous individuals in the virtual population. We previously classified the optimal-for-the-average protocol of VVVDDD as fragile because this protocol eradicates the average tumor (37), yet only 30% of individuals in our virtual cohort were successfully eradicated by this treatment (32). Importantly, fragility is a probabilistic population-level descriptor, and not an individual descriptor. While it tells us that populations that deviate somewhat from the average are less likely to behave like the average, it tells us nothing about individuals, particularly if the individuals have behavior that deviates significantly from the average (which is often the case, as shown in **Figure 1**). Though, it seems natural to hypothesize that in such a fragile region we may have to be more careful about applying a prediction for the average of a population to any one individual in that population.

We will explore that hypothesis here by looking at statistics on how individual mice respond to VVVDDD, the predicted optimal treatment protocol for the average mouse. While this protocol was effectively able to eradicate the average tumor in the population, its success across individual mice varies significantly across fitting methodologies. For the QMC-associated predictions, this protocol eradicates tumors in 75% of the individual mice (second row of the heatmaps in **Figure 4**, left). Compare this to the NLME-associated predictions, in which this protocol eradicates tumors in only 25% of the individual mice (second row of the heatmaps in **Figure 4**, right). As shown in **Figure S3**, this prediction is unchanged if we determine treatment success or failure at day 80 instead of day 30.

We can also compare response to the optimal-for-the-average protocol across methodologies. We see a qualitative agreement across methodologies (eradication or treatment failure) in only 50% of the mice (Mouse 2, 3, 5, 6). Mouse 7 is particularly interesting, as there was 95% agreement across methodologies when using *V(30*) to measure treatment success or failure, and the optimal for the average of VVVDDD is the only protocol for which treatment response differed (with QMC predicting tumor eradication, and NLME predicting treatment failure). As a further sign of caution, notice how for Mouse 1 and 4 (the cases with significant predictive discrepancies across methodologies), and Mouse 8 (intermediate case with 25% predictive discrepancies), VVVDDD eradicates the tumor with the QMC-associated parameters yet is the worst protocol that could be given (largest log(*V*(30))) for the NLME-associated parameters. This is particularly unsettling as it means the population-level optimal treatment recommendation could be the worst protocol recommendation for some individuals. This confirms our hypothesis that a population-level prediction should be applied to individuals very cautiously when in a fragile region of dosing space.

This raises the question: what if we were assessing individualized response to a protocol in a robust region of dosing space, wherein treatment response across individuals in a virtual population is statistically similar to the treatment response in the population average? In (32), we previously classified the optimal-for-the-average protocol of DDDVVV as robust in the high DC (50% greater than experimental dose), low OV (50% lower than experimental dose) region of dosing space. It was classified as robust because this protocol eradicates the average tumor, and it also eradicates 84% of the individuals in our virtual cohort (32). This probabilistic population-level assessment of robustness naturally leads to the hypothesis that in a robust region of dosing space, we may have more success with the optimal-for-the-average treatment in individual mice. We will explore that hypothesis here.

**Figure 8:**
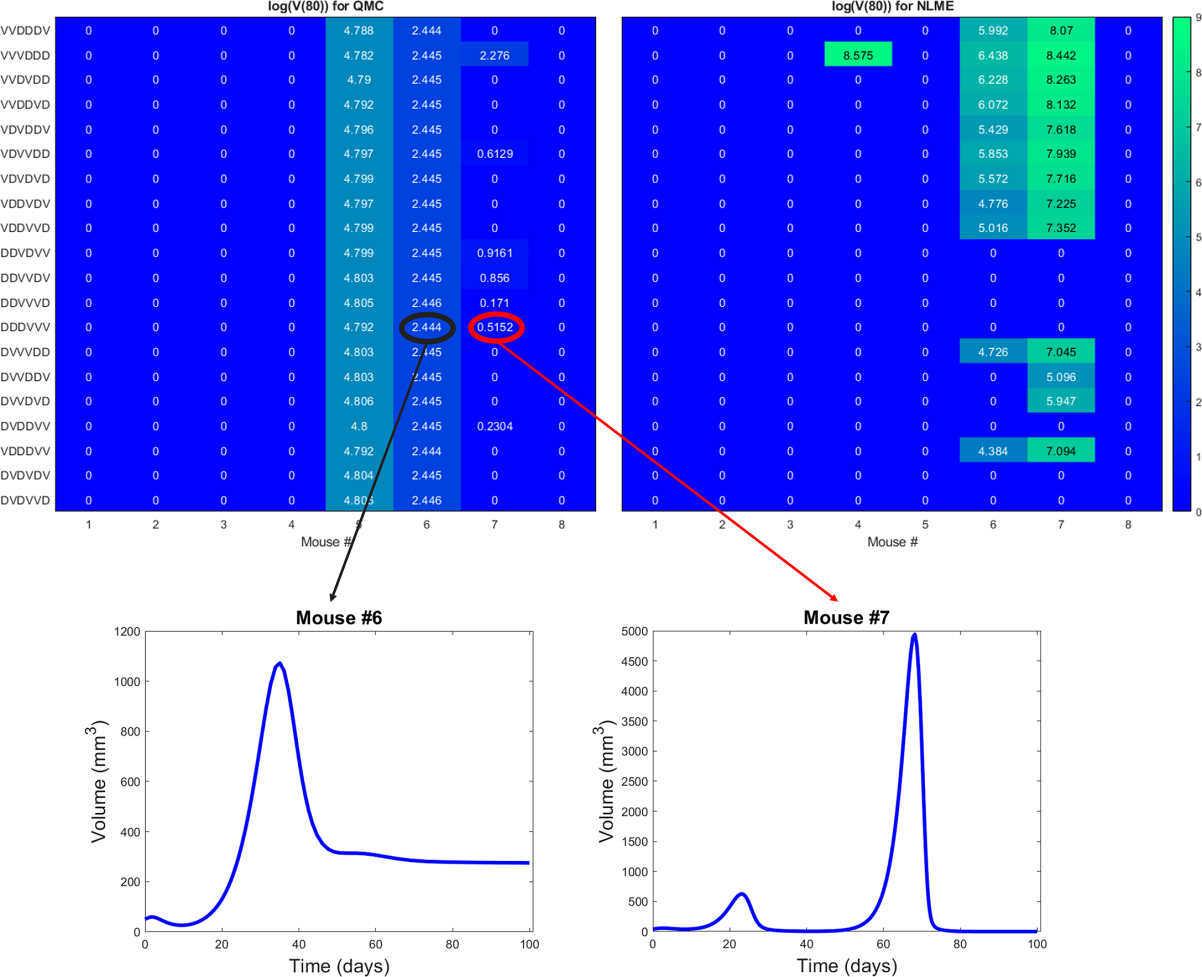
Heatmaps showing the log of the tumor volume measured at 80 days, at the high DC (50% greater than experimental dose), low OV (50% lower than experimental dose) region of dosing space. Left shows predictions if parameters are fit using QMC, and right shows NLME predictions. Inserts show time course of predicted treatment response for Mouse 6 and 7 to the optimal-for-the-average protocol of DDDVVV.

The robust population-level optimal of DDDVVV yields a successful treatment response in all eight mice for the NLME-associated parameters. This holds whether we use *V(30*), our original measure for establishing treatment success (as shown in **Figure S6**), or if we use *V(80*) as shown in **Figure 8**. This is consistent with the robust nature of this region of dosing space, as the NLME-associated parameters are less likely to wildly deviate from the average mouse due to population-level distributions constraining the value of these parameters. In comparison, the QMC-associated predictions show that only 62.5% of the individual mice are successfully treated by the optimal for the average in an 80-day window (**Figure 8**, top left). That said, if we look at the data more closely, we can see that Mouse 7 has essentially been eradicated even though 80 days was not quite long enough to drive *V(80*)<1 mm^3^, our threshold for eradication. **Figure 8** also shows that the tumor volume for Mouse 6 has stabilized. Thus, we see that the QMC-associated predictions actually agree with the optimal-for-the-average response in 75% of cases (or, 87.5% if you consider the stabilization of Mouse 6 to be a “success” rather than a “failure”).

In closing, we have confirmation of our hypothesis that there is a significant benefit to working with a robust optimal-for-the-average protocol, even in the absence of all model parameters being practically identifiable. In the presence of robustness, we predict that one could generally apply the optimal-for-the-average protocol and expect a qualitatively similar response in most individuals. While this does not mean each individual is treated with their personalized optimal protocol, this has important consequences for determining when a population-level prediction will be effective in an individual.

## 4 Discussion

In this work, we demonstrated that computational challenges can arise when using individualized model fits to make treatment recommendations. In particular, we showed that treatment response can be sensitive to the fitting methodology utilized when lacking sufficient patient-specific data. We found that for our model and preclinical dataset, predictive discrepancies can be at least somewhat explained by the lack of practical identifiability of model parameters. This can result in the dangerous scenario where an effective treatment recommendation according to one fitting methodology is predicted to be the worst treatment option according to a different fitting methodology. This raises concerns regarding the utility of mathematical models in personalized oncology when individual data is limited.

While it is well-established that more data improves parameter identifiability, here we highlight how we can identify precisely what data would improve the reliability of model predictions. In particular, we see how having a single additional measurement on the viral load or T cell count at the end of the data collection window would go a long way to reducing the predictive discrepancies across fitting methodologies (**Figure 7**). While the full benefits of this observation are not realized in a retrospective study, they could be realized in a scenario where data collection and modeling are occurring simultaneously. In this scenario, an experimentalist could collect data on a small number of individuals (like the eight mice shown in **Figure 1**). A mathematical model validated against this data can be used to identify any predictive challenges that emerge within this dataset, and what data would be needed to overcome these predictive challenges. This would inform the experimentalist of what data to collect in the next cohort of individuals in order to have more confidence in personalized treatment predictions.

When additional data is not available, an alternative option to personalization is simply treating with the population-level optimal. Here we showed the dangers of applying the optimal-for-the-average for a fragile protocol, and we demonstrated that such a one-size-fits all approach is much safer to employ for a robust optimal protocol. Therefore, even when data is lacking to make personalized predictions, establishing the robustness of treatment response can be a powerful tool in predictive oncology.

It is of note that this study uses just one mathematical model, with one set of assumptions, to reach our cautionary conclusion regarding the fitting methodology utilized and the resulting biological predictions. And this model is quite a simple one, ignoring many aspects of the immune system, and spatial aspects of immune infiltration (as done in (50), among many other references). The model used herein was chosen because it has been previously validated against the average of the available experimental data. A more complex model would be problematic here, as there is simply not the associated experimental data to validate such a model. While this study certainly does not guarantee that similar issues will arise when working with other models and datasets, it highlights the need for caution when using personalized fits to draw meaningful biological conclusions.

As we enter the era of healthcare where personalized medicine becomes a more common approach to treating cancer patients, harnessing the power of mathematical models will only become more essential. Understanding the identifiability of model parameters, what data is needed to achieve identifiability and/or predictive confidence, and whether treatment response is robust or fragile are all important considerations that can greatly improve the reliability of personalized predictions made from mathematical models.

## Data Availability Statement

Data and code availability requests can be directed to the corresponding author.

## Author Contributions

All authors substantially contributed to the analysis of the data in this work, and the drafting of the manuscript. Each author also provides approval for publication of the content.

## Funding

JG and ML acknowledge use of the ELSA high-performance computing cluster at The College of New Jersey for conducting the research reported in this paper. This cluster is funded in part by the National Science Foundation under grant numbers OAC-1826915 and OAC-1828163.

## Conflict of Interest

The authors declare that the research was conducted in the absence of any commercial or financial relationships that could be construed as a potential conflict of interest.

## Publisher’s Note

All claims expressed in this article are solely those of the authors and do not necessarily represent those of their affiliated organizations, or those of the publisher, the editors and the reviewers. Any product that may be evaluated in this article, or claim that may be made by its manufacturer, is not guaranteed or endorsed by the publisher.
