# Supplementary material for: From Fitting the Average to Fitting the Individual: A Cautionary Tale for Mathematical Modelers
Source: Front Oncol. 2022 Apr 28;12:793908. doi: 10.3389/fonc.2022.793908 (PMC9097280; doi:10.3389/fonc.2022.793908)
Supplement: Supplementary file 1 [file DataSheet_1.pdf]

Supplementary Material

1 SUPPLEMENTARY METHODS

Algorithm 1 Independent Fitting Algorithm

Require: Time-course volumetric data per mouse
  for each mouse i **do**
    Generate parameter set p, a set of 10^6 12-dimensional Sobol points
    Scale each point to be in biologically restricted range
    for each parameter set j **do**
      solve ODEs in eqns. (1)-(5)
      Compute $\zeta_i(p_j)$
    end for
    $p_{min}(i) \leftarrow \arg\min_{p(j)} \zeta_i(p_j)$
    while Relative change in $\zeta < 10^{-5}$ over last 5 accepted changes **do**
      $\alpha = \mathcal{O}(p_{min}(i))$
      $r =$ random number in the range $[-10^{\alpha-3}, 10^{\alpha-3}]$
      $p_{temp} \leftarrow p_{min}(i) + r$
      Solve ODEs in eqns. (1)-(5) at parameter set p_{temp}
      Compute $\zeta_i(p_{temp})$
      $\Delta = \zeta_i(p_{temp}) - \zeta_i(p_{min}(i))$
      if $\Delta < 0$ **then**
        $p_{min}(i) \leftarrow p_{temp}$
      end if
    end while
  end for

Parameter	Minimum	Maximum
r	0.27	0.41
β	0.87	1.11
δ_V	1.15	4.60
α	1.50	6.00
δ_I	0.50	2.00
k_0	1.00	4.00
χ_D	3.46	12.05
c_T	0.53	8.04
c_{kill}	0	1.19
δ_T	0.17	0.70
δ_D	0.17	0.70
$U_0(i)$	$0.5U_i(0)$	$2U_i(0)$

Table S1. Minimum and maximum value of each parameter for quasi-random Monte Carlo step of independent fitting algorithm. The range for the initial condition $U_0(i)$ is set depending on the initial volume of Mouse i in the experimental data.

2 SUPPLEMENTARY FIGURES

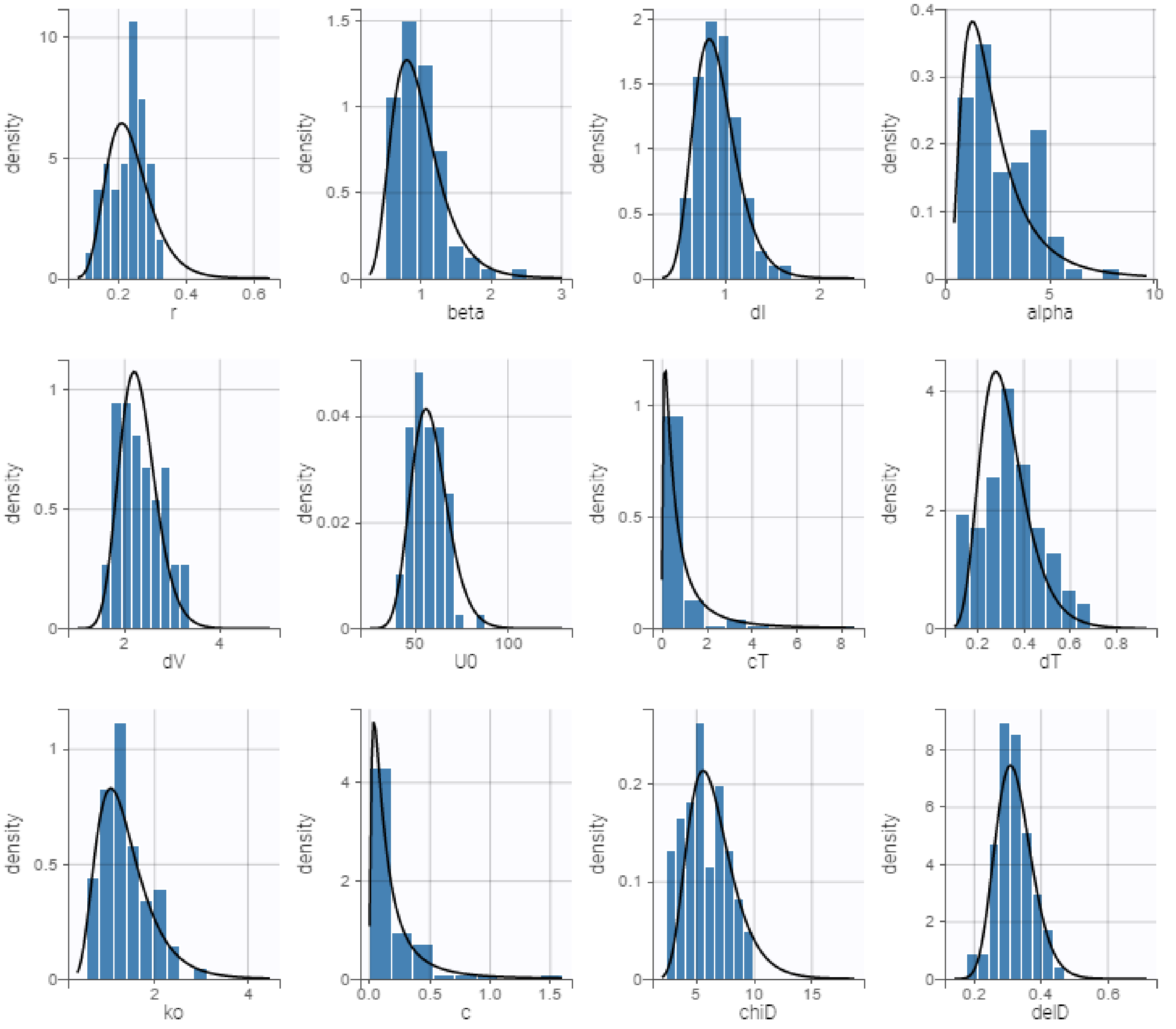

Figure S1. Estimated parameter distributions from Monolix's implementation of NLME. Each parameter is assumed to be lognormally distributed. The blue bars in each graph represent the empirical distribution of the parameter estimation and the black line represents the theoretical distribution.

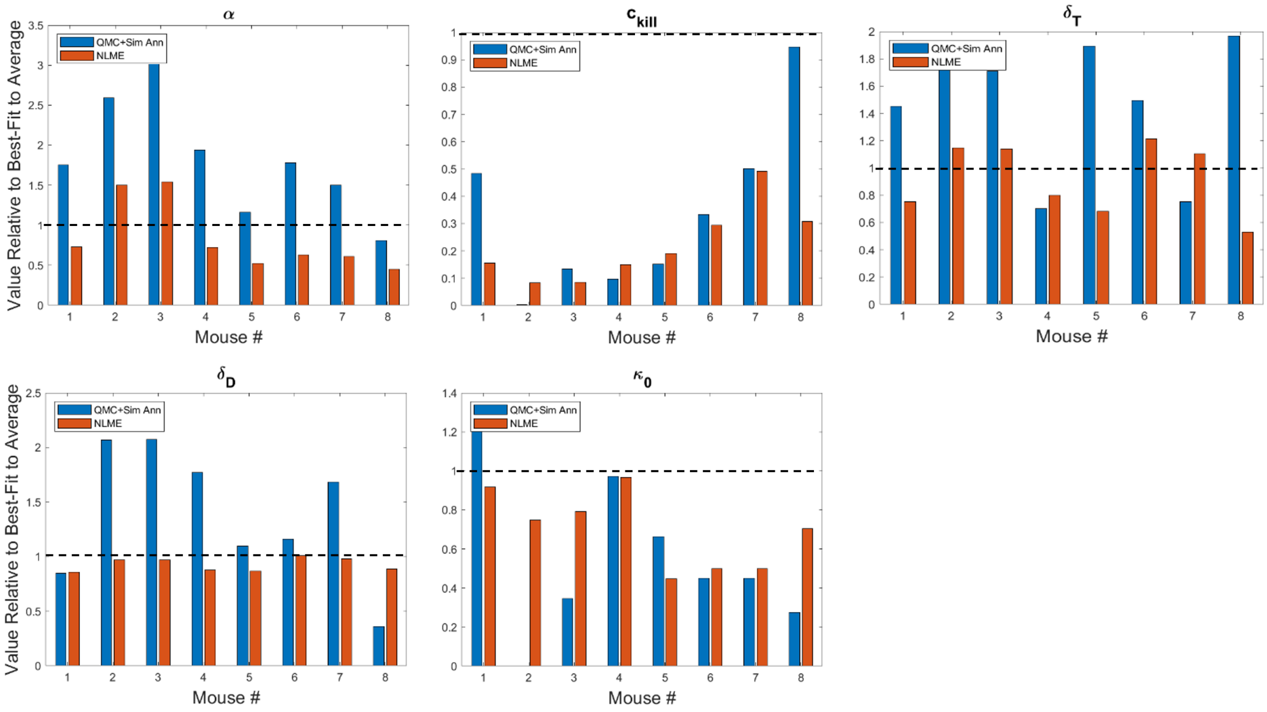

Figure S2. Best-fit value of number of viruses released by lysed cell α, the cytotoxicity enhancement term due to immunostimulants c_{kill}, T cell decay rate δ_T, DC decay rate δ_D, and default cytotoxicity rate of T cells κ_0. The best-fit values are shown for each mouse and are presented relative to the best-fit value of the parameter in the average mouse [1]. Therefore, a value of 1 means the parameter value is equal to that in the average mouse (shown in the dashed black line), less than 1 is a smaller value, and greater than 1 is a larger value.

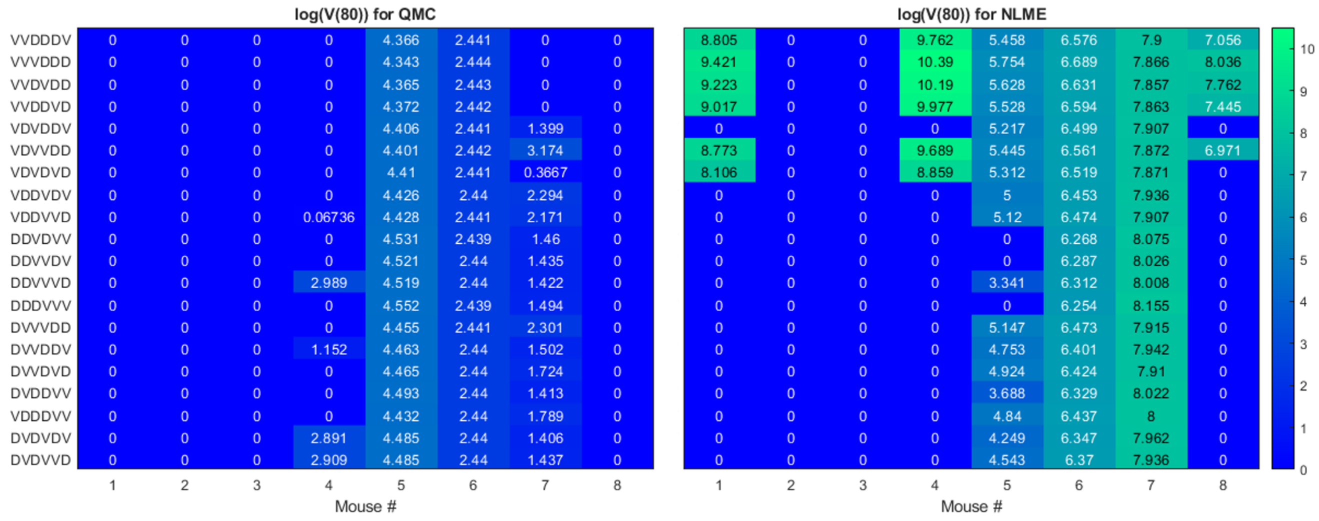

Figure S3. Heatmaps showing the log of the tumor volume measured at 80 days, at the OV and DC dose used in [2]. Left shows predictions when parameters are fit using QMC and right shows NLME predictions. Compare to heatmap in Fig. 4 which shows the log of the tumor volume 50 days earlier.

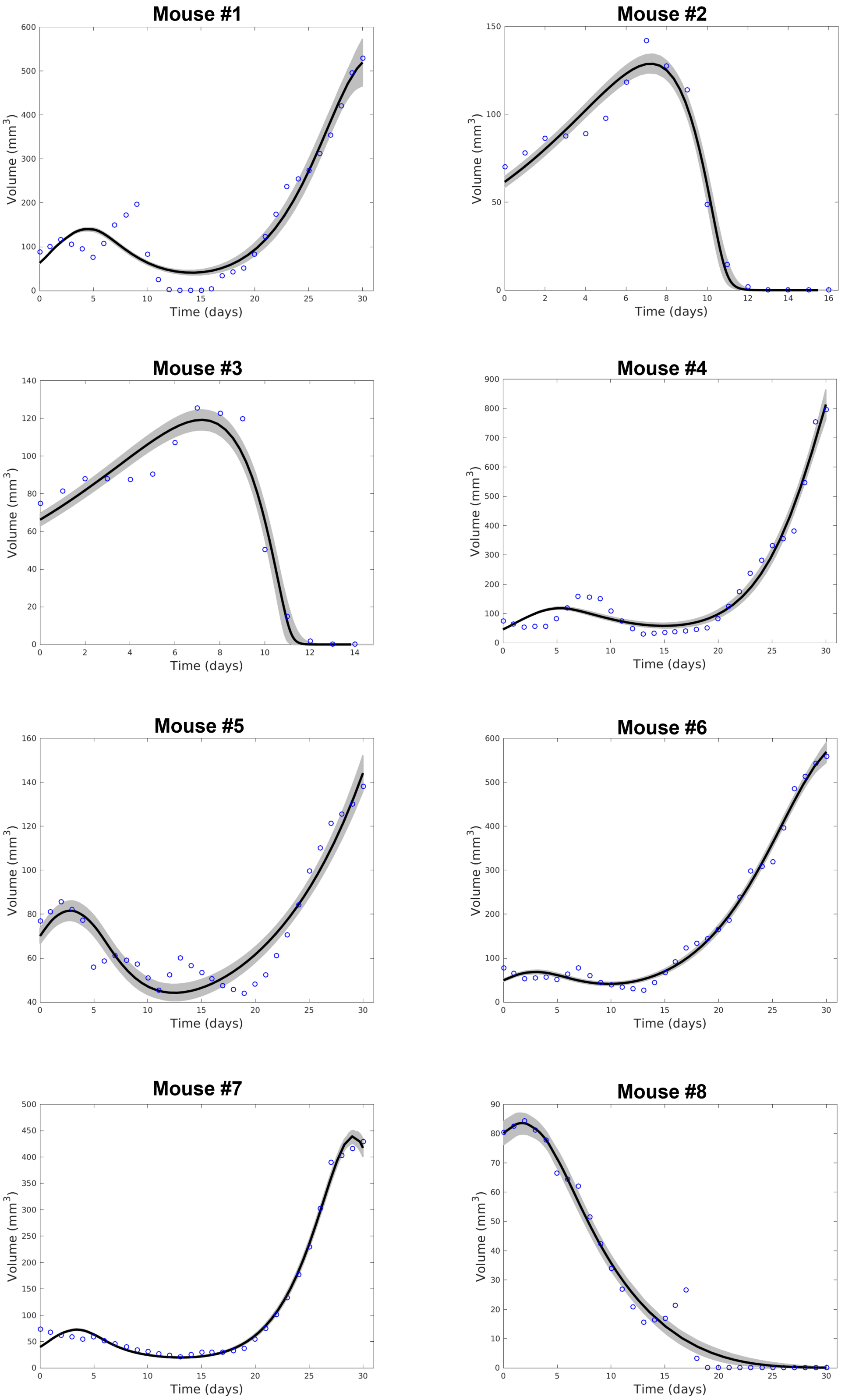

Figure S4. Optimal QMC fit (black) with suboptimal QMC fits (grey) to experimental data (blue outlined circles) for which the goodness-of-fit metric is within 10% of optimal.

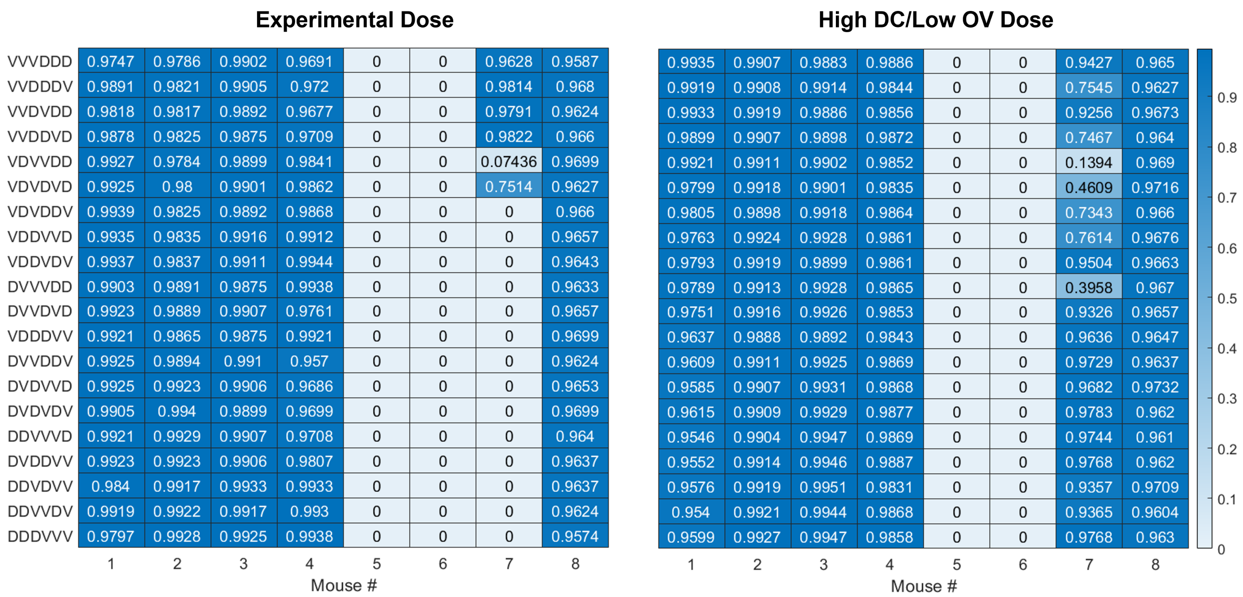

Figure S5. Probability of an effective treatment over the suboptimal parameter sets per mouse (for QMC-associated parameters), across the 20 treatment protocols. The experimental dose is shown on the left, and the high DC/low OV dose is shown on the right.

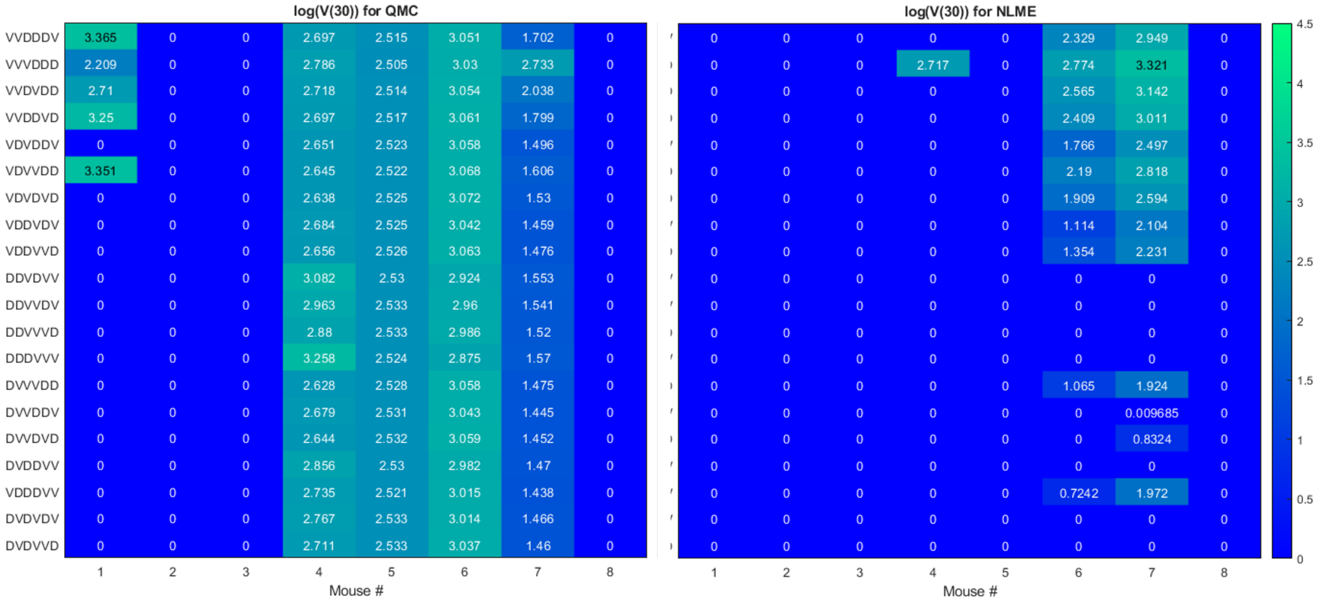

Figure S6. Heatmaps showing the log of the tumor volume measured at 30 days, at the high DC (50% greater than experimental dose), low OV (50% lower than experimental dose) region of dosing space. Left shows predictions if parameters are fit using QMC and right shows NLME predictions. Compare to heatmap in Fig. 8 which shows the log of the tumor volume 50 days later.

REFERENCES

[1] J.L. Gevertz and J.R. Wares. Developing a minimally structured model of cancer treatment with oncolytic viruses and dendritic cell injections. *Comp. Math. Meth. Med.*, 2018:8760371, 2018.

[2] J.-H. Huang, S.-N. Zhang, K.-J. Choi, I.-K. Choi, J.-H. Kim, M. Lee, H. Kim, and C.-O. Yun. Therapeutic and tumor-specific immunity induced by combination of dendritic cells and oncolytic adenovirus expressing IL-12 and 4-1BBL. *Molecular Therapy*, 18:264 –274, 2010.
